# A beginner’s view of end of life care on German intensive care units

**DOI:** 10.1186/s12871-022-01684-8

**Published:** 2022-05-18

**Authors:** Timur Sellmann, Muhammad Abu Alneaj, Dietmar Wetzchewald, Heidrun Schwager, Christian Burisch, Serge C. Thal, Tienush Rassaf, Manfred Weiss, Stephan Marsch, Frank Breuckmann

**Affiliations:** 1 Department of Anaesthesiology and Intensive Care Medicine, ev. Bethesda Krankenhaus, Duisburg, Germany; 2 Department of Anaesthesiology I, University of Witten/ Herdecke, Witten, Germany; 3 Institute for Emergency Medicine, Arnsberg, Germany; 4 State of North Rhine-Westphalia / Regional Government Düsseldorf, Düsseldorf, Germany; 5 Department of Anaesthesiology, HELIOS University Hospital, Wuppertal, Germany; 6 Department of Cardiology and Vascular Medicine, West German Heart and Vascular Center Essen, University Duisburg-Essen, Essen, Germany; 7 Clinic of Anaesthesiology and Intensive Care Medicine, University Hospital Medical School, Ulm, Germany; 8 Department of Medical Intensive Care, University Hospital, Basel, Switzerland

**Keywords:** Advanced care planning, Critical care, Education, medical, graduate, Palliative care, Prognosis, Quality of life, Surveys and questionnaires, Terminal care

## Abstract

**Background:**

Little is known about importance and implementation of end-of-life care (EOLC) in German intensive care units (ICU). This survey analyses preferences and differences in training between “medical” (internal medicine, neurology) and “surgical” (surgery, anaesthesiology) residents during intensive care rotation.

**Methods:**

This is a point-prevalence study, in which intensive care medicine course participants of one educational course were surveyed. Physicians from multiple ICU and university as well as non-university hospitals and all care levels were asked to participate. The questionnaire was composed of a paper and an electronic part. Demographic and structural data were prompted and EOLC data (48 questions) were grouped into six categories considering importance and implementation: category 1 (important, always implemented), 2 (important, sometimes implemented), 3 (important, never implemented) and 4–6 (unimportant, implementation always, sometimes, never). The trial is registered at the “Deutsches Register für klinische Studien (DRKS)”, Study number DRKS00026619, registered on September 10th 2021, www.drks.de.

**Results:**

Overall, 194/ 220 (88%) participants responded. Mean age was 29.7 years, 55% were female and 60% had scant ICU working experience. There were 64% medical and 35% surgical residents. Level of care and size of ICU differed significantly between medical and surgical (both *p* < 0.001). Sufficient implementation was stated for 66% of EOLC questions, room for improvement (category 2 and 3) was seen in 25, and 8% were classified as irrelevant (category 6). Areas with the most potential for improvement included prognosis and outcome and patient autonomy. There were no significant differences between medical and surgical residents.

**Conclusions:**

Even though EOLC is predominantly regarded as sufficiently implemented in German ICU of all specialties, our survey unveiled still 25% room for improvement for medical as well as surgical ICU residents. This is important, as areas of improvement potential may be addressed with reasonable effort, like individualizing EOLC procedures or setting up EOLC teams. Health care providers as well as medical societies should emphasize EOLC training in their curricula.

**Supplementary Information:**

The online version contains supplementary material available at 10.1186/s12871-022-01684-8.

## Background

End-of-life care (EOLC) in Intensive Care Units (ICU) has gained special attention during the Corona pandemic and is under discussion worldwide. Both national and international intensive care medicine societies are requesting guidelines and/or recommendations concerning this topic [1]. Evidence regarding a national framework for EOLC is rare. Following two publications of Weiss et al investigating anaesthesiologist-led ICUs, we revisited the problem of a profound discrepancy between current practice and attributed importance identified therein in order to gain more information on the topic and add more evidence to existing literature [2, 3]. We sought to investigate attitudes on a subordinate, resident level, not only on anaesthesiologist-led, but also on medical, surgical, neurological, and interdisciplinary ICUs. Additionally, with a growing number of physicians from different cultural backgrounds caring for patients from a variety of ethnic and cultural backgrounds in German ICUs, the complexity of EOLC is increasing, especially concerning the withdrawal of life-sustaining treatment while ensuring the alleviation of suffering [4]. Furthermore, there may be varying attitudes towards EOLC between “medical” (i.e. internal medicine and neurology in this context) and “surgical” (anaesthesiology and surgery) intensive care medicine. Prior studies found a strong influence of providers’ personal views on principles of EOLC [5]. Staff surveys might add information about importance and degree of implementation and the perceived need for action. As there is growing interest in this topic, we focused on educational aspects to ascertain the expectations of residents from different medical backgrounds. The aim of our study was to obtain further knowledge about underlying basic EOLC requirements, existing EOLC practices, importance and relevance of such structures as well as personal expectations and requests in order to define future need for educational action on EOLC in ICUs for residents completing their intensive care rotation, irrespective of personal subspecialties..

## Methods

This is a point-prevalence survey in order to gain information about the *status quo* and need for propaedeutics as well as in situ action for EOLC in ICU.

### Participants and setting

The Working Group on Intensive Care Medicine, Arnsberg, Germany (http://www.aim-arnsberg.de), organises specific educational courses six times per year for physicians from all over Germany and German-speaking countries, who are at the beginning of their obligatory intensive care training as part of their residency program. The educational courses are comprised of 64 hours of continuing medical education, consisting of lectures (including a one-hour lecture on palliative care) and practical drills. Within this course format, 1300–1400 residents are trained annually.

The majority of the participants consists of 2nd and 3rd year residents in internal medicine, anaesthesia, surgery and neurology. Attendees of one course were offered to participate in a voluntary, split televoting and paper-based survey on EOLC. In order to find out more about differing attitudes related to specialty, participants were grouped in two major groups: “medical” (internal medicine and neurology) and “surgical” (anaesthesiology and surgery).

### Ethics approval and consent to participate

Prior to distribution of the survey, participants were informed that the data collected was to be anonymized. Therefore, the regional ethical committee (Ethik-Kommission der Ärztekammer Westfalen-Lippe und der Westfälischen-Wilhelms Universität Münster) waived the need for approval and especially for obtainment of written informed consent (reference: 2019–401-f-S). The trial is registered at the “Deutsches Register für klinische Studien (DRKS)”, Study number DRKS00026619, registered on September 10th 2021, www.drks.de. No written informed consent was therefore collected. All methods were carried out in accordance with good scientific practice.

### Study design

#### Questionnaire

The survey was modified from prior publications by Weiss et al. [2, 3] consisting of 78 questions overall, of which 30 were related to structural data (see supplemental material, Appendix A) and 48 were related to EOLC in the ICU of the appointed hospitals. The structural questionnaire (Appendix A) included the following aspects: Hospital category, level of care, hospital sponsor, total number of beds and intensive care beds, treatment focus, number of ICU and Intermediate Care (IMC) patients treated annually, number of physicians and senior physicians working in the participating ICU and IMC, number of ICU doctors and nurses additionally qualified in palliative care, physician: patient and nurse: patient ratio respectively, availability of palliative care physicians, health care chaplaincy and psychologists.

The EOLC related questions included prognostic scores, reporting of individual patient outcomes, goals of care, patient autonomy, standard operating procedures, quality management, limitations of life-sustaining therapy, nursing aspects and concepts of care for dying patients. Questionnaire and questions can be found in Table 2. For each item, participants were asked to state importance and current implementation. Importance was rated binary as “important “or “unimportant”. Current implementation was rated on a modified three-point Likert scale as “yes, always”, “often, sometimes” or “no, never”. Considering implementation, importance and resulting relevance, we determined six subgroups (according to [3]):

**Table:** 

	Importance	Implementation	Relevance
**Category 1**	important	always	sufficient
**Category 2**	important	sometimes	improvable
**Category 3**	important	no, never	deficient
**Category 4**	not important	always	redundant
**Category 5**	not important	sometimes	misallocated
**Category 6**	not important	no, never	irrelevant

Assignment to one of the six categories was based on a simple majority vote for each response. Categories implying room for improvement were 2, 3, 4 and 5 and for illustration, blob-o-grams were used. Category 1 (“sufficient”) and category 6 (irrelevant”) were not specifically evaluated further.

### Data analysis

Primary outcome variable: To collect data on the “*status quo*” of underlying structure and current EOLC practices in ICUs from a “medical” and “surgical” point of view. Secondary outcome measures include actual structural premises and actual EOLC practices, subdivided into actually conducted, deemed important or relevant, and derived need for action in the future. Finally, the integration of expectations of course participants into future educational activities should be discussed.

### Statistics

This is a primarily descriptive data-analysis aiming to generate hypotheses for future studies on EOLC on ICU. Data was proved for distribution. We used unpaired Student’s t-test, Fisher’s exact test and Pearson’s Chi Square test where appropriate. A *p* < 0.05, two-tailed, was determined as statistically significant. Data are presented as range and means with standard deviation. For statistical analysis, PSPP was used (freeware; https://www.gnu.org/software/pspp/).

All data generated or analyzed during this study are included in this article and its supplementary information files. For additional dataset request are available from the corresponding author on reasonable request.

## Results

The study was undertaken in 2019. Of 220 questionnaires, 195 were returned and 194 were complete and evaluable (88.2%).

### Demographic data

Demographic data were available for 173 participants. “Medical” residents represented 67% and “surgical” residents 33%. Mean age was 30 years, 55% were female. More than half (56%) of the participants were second- or third-year residents. Previous ICU working experience existed in 60%, of which 91% had less than 1 year of experience. Additional demographic data and differences between “medical” and “surgical” residents are depicted in Table 1.

**Table 1:** Demographic and structural data of course participants

		All participants		Medical		Surgical		***p*** =
**Age** **(*n*** **= 173)**		**n =**	**years**	**n =**	**years**	**n =**	**years**	
		173	29.7	115	29.5	58	30.2	0.427^$^
**Gender** **(*n*** **= 186)**		**n =**	**%**	**n =**	**%**	**n =**	**%**	
	m/f/nb	83/102/1	44.6/54.8/0.6	55/66/1	45.1/54.1/0.8	28/36/0	43.8/56.3/0	0.749^¶^
**Doctoral thesis** **(*n*** **= 212)**	y/n	70/142	33.0/67.0	48/92	34.3/65.7	22/50	30.6/69.4	0.645^†^
**Specialization** **(*n*** **= 215)**	y/n	10/205	4.7/95.3	8/133	5.7/94.3	2/72	2.7/97.3	0.500^†^
**Emergency physician** **(*n*** **= 215)**	y/n	9/206	4.2/95.8	4/139	2.8/97.2	5/67	6.9/93.1	0.171^†^
**ICU work experience** **(*n*** **= 215)**	y/n	127/88	59.1/40.9	90/51	63.8/36.2	37/37	50/50	0.058^†^
**Hospital level of care** **(*n*** **= 211)**	Max^a^/Non-Max^b^	84/127	39.8/60.2	44/96	31.4/68.6	40/31	56.3/43.7	0.001^†^
**Number of ICU beds** **(*n*** **= 212)**	≤14/> 14	136/76	64.2/35.8	101/38	72.7/27.3	35/38	47.9/52.1	0.001^†^
**Resuscitation attained** **(*n*** **= 214)**	≤10/> 10	155/59	72.4/27.6	102/39	72.3/27.7	53/20	72.6/27.4	1.000^†^
**Resuscitation lead** **(*n*** **= 211)**	≤10/> 10	194/17	91.9/8.1	133/8	94.3/5.7	61/9	87.1/12.9	0.104^†^

n = indicating total number of answers; since participation was completely voluntarily, numbers might deviate

*m* male, *f* female, *nb* nonbinary; ^a^ university hospitals and level three hospitals; ^b^ hospitals below level 3; ^$^t-Test; ^¶^Chi Square (Pearson); ^†^Fisher’s exact test

### Structural data

Detailed structural data are displayed as supplemental material. Of all structural data, only “hospital level of care” and “number of ICU beds” reached statistical significance (both *p* < 0.001) with a significant higher number of “surgical” residents from maximum care providers attending the course (Table 1).

### EOLC on ICU

**Fig. 1:**
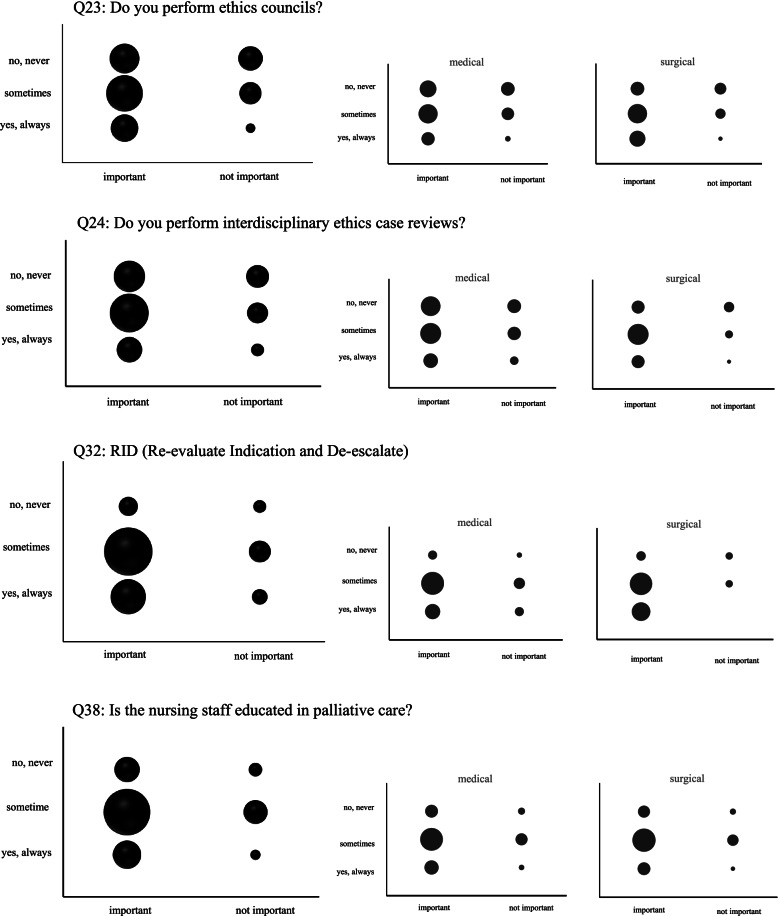
Interdisciplinary distribution of category 2 (“improvable”) questions. Figure 1 shows a score cloud according to the corresponding number of mentions, first for the total collective, then for the “surgical” and “medical” subpopulations. From a purely visual point of view, there are no significantly noticeable differences in the different response behaviour

Of 48 question items related to EOLC in ICU, implementation was classified as “sufficient” (category 1) in 67%, four items (8%) were judged as “improvable” (category 2), eight (17%) were deemed “deficient” (category 3) and another four questions were assessed as “irrelevant” (category 6). There were no category 4 (“redundant”) or category 5 (“misallocated”) items. There was no significant difference in the response behaviour between “medical” and “surgical” residents. Data on all questions, response patterns and categorisation are presented in Table 2. The four questions implying room for improvement were further analysed using blob-o-grams. The results are presented in Fig. 1.

**Table 2:** EOLC on ICU items and interdisciplinary response pattern

		Category	Medical	Surgical	P* =
**Prognosis and outcome (Q 1–6)**					
Q1	Scores, such as SAPS II or SOFA, to estimate a patient’s individual prognosis? (ICU stay < 24 h?)	**1**	25/125	15/63	0.574
Q2	Scores, such as SAPS II or SOFA, to estimate a patient’s individual prognosis? (With ICU stay > 24 h?)	**1**	46/119	18/59	0.322
Q3	Do you receive outcome data regarding long-term survival after hospital discharge?	**3**	74/120	34/64	0.275
Q4	Do you receive outcome data from patients discharged to other hospitals or rehabilitation centers?	**3**	43/119	28/63	0.338
Q5	Do you receive outcome data from patients discharged home?	**3**	71/121	39/63	0.752
Q6	Do you use outcome data from your hospital for your decisions?	**3**	41/111	19/59	0.614
**Goals of care (curative versus palliative) (Q 7–17)**					
Q7	Do you use principles of palliative care?	**1**	59/120	25/62	0.276
Q8	Do you address goals of care within 72 h of ICU admission?	**1**	91/112	48/63	0.441
Q9	Do you discuss goals of care and prognosis with patients and families?	**1**	107/117	59/63	0.773
Q10	Do you document the items and results of these conversations with patients?	**1**	93/114	54/63	0.536
Q11	Do you document the items and results of these conversations with relatives?	**1**	92/114	52/61	0.536
Q12	Do you discuss indications in an interdisciplinary manner?	**1**	71/118	42/63	0.424
Q13	Do you discuss whether goals are achievable?	**1**	80/117	47/64	0.502
Q14	Do you discuss ineffective therapy?	**1**	82/115	43/63	0.733
Q15	Do you establish feasible and realistic treatment goals?	**1**	89/113	54/63	0.316
Q16	Do you discuss whether a desirable quality of survival is achievable?	**1**	73/117	36/63	0.525
Q17	Do you decide on and document to allow natural death (AND)?	**1**	66/115	37/61	0.749
**Patient autonomy (Q 18–24)**					
Q18	Do you document the assumed consent of the patient?	**1**	88/117	49/63	0.855
Q19	Do you document conversations with relatives regarding the assumed consent of the patient?	**1**	1/123	0/65	0.814
Q20	Do you document conversations with the patients regarding their priorities regarding their way of life, their perceptions of quality of live, and their wishes for the future?	**1**	60/116	30/62	0.753
Q21	Do you have guidelines for dealing with delicate wishes of patients?	**3**	56/121	25/61	0.530
Q22	Do you have an ethics committee?	**1**	43/115	33/62	0.056
Q23	Do you perform ethics councils?	**2**	38/115	22/61	0.739
Q24	Do you perform interdisciplinary ethics case reviews?	**2**	37/121	29/63	0.052
**Standard operating procedures (SOPs), quality management (Q 25–27)**					
Q25	Do you have SOPs for psychosocial problems?	**3**	51/122	25/61	1.000
Q26	Do you have SOPs for spiritual problems?	**6**	48/121	33/60	0.058
Q27	Do you have a room for taking farewell?	**1**	60/118	37/62	0.275
**Which changes in goals of care do you execute in these instances? (Q 28–35)**					
Q28	Continuation and escalation of therapy with all consecutive life-sustaining activities?	**1**	53/113	37/64	0.211
Q29	Change in goals of care, adjustment of therapy to the new goals, usually by limitations of care?	**1**	80/110	42/64	0.391
Q30	DNR (Do Not Resuscitate)	**1**	79/112	47/63	0.603
Q31	DNE (Do Not Escalate)	**1**	62/114	42/64	0.157
Q32	RID (Re-evaluate Indication and De-escalate)	**2**	59/114	29/62	0.533
Q33	CTC (Comfort Terminal Care)	**1**	53/114	21/62	0.113
Q34	Is the decision to changing goals of care authorized by a physician, communicated during handover of duty, checked daily and documented in the patient chart / patient data management system?	**1**	79/113	48/61	0.283
Q35	Do you have a checklist” Items for intensive care medicine for individual changes in treatment goals”?	**3**	50/114	31/58	0.260
**Nursing aspects (Q 36–38)**					
Q36	Do you integrate nurses’ opinions?	**1**	81/112	38/62	0.173
Q37	Do you implement palliative care concepts, such as adaption of oral care, noise, light, basal stimulation?	**1**	57/109	26/60	0.335
Q38	Is the nursing staff educated in palliative care?	**2**	54/109	30/57	0.745
**Concepts of care in the terminal phase (Q 39–48)**					
Q39	Do you use SOPs for EOL?	**3**	35/116	16/56	0.861
Q40	Do you do an appraisal of the initial situation?	**1**	61/112	31/60	0.751
Q41	Is there care for others, such as relatives or the primary care physician, once the patient has died?	**1**	44/116	21/59	0.869
Q42	Do you use the Liverpool pathway of care?	**6**	60/92	35/49	0.572
Q43	Do you administer diaries of patients?	**6**	69/106	39/62	0.868
Q44	Do you administer diaries of relatives?	**6**	81/114	44/63	0.865
Q45	Do you involve relatives to attend when death occurs?	**1**	99/109	52/65	0.062
Q46	Do you offer attendance by psychologists, social workers, spiritual care?	**1**	77/112	46/64	0.734
Q47	Do you consider intercultural aspects?	**1**	67/111	31/64	0.155
Q48	Are visiting hours handled flexible according to the needs of the Relatives?	**1**	81/112	39/64	0.132

Category 1 (“sufficient”; important, always implemented), Category 2 (“improvable”; important, sometimes implemented), Category 3 (“deficient”; important, never implemented), Category 6 (“irrelevant”; not important; never implemented); *chi square test

The items characterizing the most urgent need for improvement (category 3) referred to “prognosis and outcome”, “patient autonomy”, “standard operating procedures and quality management”, “preparation of health care directives” and “concepts of care in the terminal phase”. There was no category 3 aspect within “goals of care” or “nursing aspects”.

## Discussion

Our work provides an interdisciplinary view on a non-specialist level adding valuable insight into the attitudes and needs of residents towards EOLC in German ICUs and is one of few studies dealing with this topic in general [6–8]. Although 2/3 of EOLC questions were classified as “sufficiently implemented” still 25% of issues addressed showed room for improvement.

Medical guidelines or treatment recommendations are usually developed by experts; when it comes to bedside care, residents are also involved in patients’ treatment, and they may experience personal dilemmas or conflicts by not knowing the guidelines, or having difficulties how to implement them into daily practice or even disagree with the guidelines on different levels. A field of growing interest is EOLC on ICU, triggered by the Corona pandemic and its associated shortfalls [1].

### Demographic data

An overall response rate of 88% reflects a high level of interest in the topic among the study population, that was equally distributed in terms of demographic data and work experience. It was most likely co-triggered by the prospect of a small gift for participation > 90%. About 90% had less than 1 year of ICU working experience, so the term “novices” is justified in this context. Interdisciplinary ICUs, often anaesthesiologically managed, have been assigned to “surgical” ICUs in our context, since it can be assumed that, if at all, surgical ICU patients are regularly treated in surgical or anaesthesiological, but not in medical ICUs. Regarding own anonymous and unpublished data, available from the authors on request, the 2:1 ratio of “medical” and “surgical” residents is representative for this course format.

### Structural data

The higher number of “surgical” residents from maximum care providers explains the higher share of tertiary care facilities, which in turn frequently provide more ICU beds e. g. owing to specialization and disease severity of the patients treated [9], so basically seen in this way, one might cause the other.

### EOLC in ICU

Overall, 67% of EOLC items were characterized as sufficiently implemented by the respondents. Room for improvement was indicated in 25% (8% “improvable”, 17% as “deficient”). Only a small proportion (8%) was classified as “irrelevant”, which could indicate a high level of interest in the topic itself. Our data amend studies regarding ICU specialists’ attitude toward the topic [2, 3], create a more comprehensive view of the *status quo* and reveal further optimization potential. Since our study population consisted of 2/3 internists and neurologists, our data give valuable insight into “medical” intensive care medicine. However, it is a genuine duty of all intensivists to constantly aim for high-quality EOLC, which, amongst others, is achieved by continuous training, implementing therapeutic goals, mentoring younger colleagues and assisting the ICU team, e. g. in order to make dignified dying in ICU more acceptable [10]. Of note, 50% of the items assessed as important, but unsatisfactorily implied in clinical practice EOLC data were in the category “prognosis and outcome” (Q 1–7). This is an observation comparable to experts [3], albeit to a lesser extent in our data. Feedback on outcome data in absence of adequate prediction models might be helpful and could be appreciated as very important for individual decision making (Q 4–6). Ideas already expressed in the context to improve EOLC include interdisciplinary rounds, advanced health care planning, and structured feedback on outcome data. In addition to medical indications, the patient’s will is an indispensable prerequisite for initiating or conducting a therapy. With regards to intensive care in particular, it cannot always be ensured that patients receive the care they would want if they were fully informed about their prognosis and likely outcome, leaving a certain degree of uncertainty. This may lead to a considerable variability between hospitals and physicians in terms of EOLC in ICU in combination with a lack of compelling evidence or professional consensus for specific approaches [11, 12]. Physicians triggered by limited ICU resources, seek prediction tools to facilitate allocation of ICU beds to patients which might benefit best [1, 13]. There are several triage outcome scores providing data on whom to refuse or whom to admit on ICU [14, 15]. Factually, once a patient is being discharged from a German ICU, nor regular follow-up is implemented and uncertainty about outcome might occur. Outcome data on mid- and long-term survival and functional status would probably be helpful especially for younger ICU residents. In Germany, for example, this information could be provided by the patients’ general practitioner at certain time points (3,6 or 12 months after discharge) or ICU teams could seek this data themselves.

Further issues classified as “deficient” regarded delicate wishes (Q21) and standard operating procedures (SOP) for patient’s psychosocial problems (Q25), both being defined as “ethical problems” in a broader sense. Experienced intensivists saw significantly more potential in this context assessing four further questions, all in the fundamental context of ethical considerations [3]. Needs arise from the feeling of deficiency and the simultaneous desire to eliminate it. Considering the prevailing short rotation time of < 6 months on average (if at all), a need for more extensive implementation has probably not developed yet and experts may have undergone a development process. It is a well-known phenomenon that intensive care medicine is often caught in the middle of ethical issues, which has been further exacerbated by the Corona pandemic. Appropriate handling of ethical issues is an important key to high-quality intensive care for patients and staff, always focusing on the human being. These problems and potential solutions have been addressed before, e. g. improving individual ethic competency by intensified medical education [16]. However, the mere existence of an ethics committee does not solve the wider issue of ethics in this context. German and Canadian guidelines and other approaches may offer help to shift to a “sufficient” implementation [17, 18]. Again, “not prioritizing life extension over good death” is of major importance for rethinking EOLC on ICU [19].

Other deficiencies identified were lack of” Items for individual changes in treatment goals” (Q35) and “SOP for EOL” (Q39), both addressable by checklists. The German interdisciplinary association of intensive care medicine (DIVI) recently published a document enabling evaluation, documentation and changing on demand the goals of care on a daily basis if needed [20]. On surgical ICUs, the only factors positively triggering DNR (“do not resuscitate”) orders are derived from past medical history [21]. Although the WELPICUS study found an agreement of 95% in key EOL issues and terminology worldwide [22], international, national, regional and even in-hospital existing varieties, often caused by individual views and local policies, might impose barriers of unknown extent [3]. In order to further advance terminology adaptation, suggestions for renaming, such as using “time-limited trial” instead of “no escalation of treatment” have been made [23]. It should always be made unmistakably clear to patient and team, that changing goals of care towards “withholding” or “withdrawing” does not imply termination of medical care [24]. “Withholding” or “withdrawing” are not equivalent terms for “giving up” [19, 25].

In contrast to category 3 (“deficient”), category 2 items were classified as “improvable”, since an occasional implementation was stated. Two of the items belonged to “patient autonomy”, one item each to “change in goals of care” and “nursing aspects”, respectively. The subsequent graphical breakdown of the response pattern into “blob-o-grams” including a split-up between “medical” and “surgical” residents enabled a more precise distribution and trending towards category 1 (“sufficient”) or category 3 (“deficient”) (Fig. 1). It was interesting to see that items concerning “ethics” were, although deemed important, obviously generally less implemented than the other two items “re-evaluate indication” and “education in palliative care”. The subdivision into “medical” and “surgical” did not reveal any specific information. The provision of a clinical ethics committee is a component of various structural characteristics assessments, so it is fundamentally astonishing that a need is still seen [25]. Not only the mere existence, but the activity of such a body must be given so that the deficits mentioned here can be addressed and remedied by it. The problems of re-evaluation of indication can easily be addressed once recognized; further training in palliative care is, though desirable, harder to execute due to additional financial burden.

### Strengths and limitations

Due to its wide range addressing over 1300 physicians annually, we judged this course format generally to be suitable to analyse residents’ ideas, attitudes and opinions on EOLC in German ICUs. The high return rate of 88% valid questionnaires allows valuable insight into the interdisciplinary point of view of novice intensivist from “medical” and “surgical” disciplines in Germany. The share of 17% of unsatisfactorily implemented EOLC on ICU items still shows potential for action, partly analogous, partly divergent to already known deficits. Contrary to prior data, we did not experience barriers to scientific investigations driven by economic competition [3]. As our survey exclusively addressed physicians, results cannot be transferred to other specialties, professions, or persons affected by EOL decisions, such as nurses, palliative care experts, or families. Even in the small group of ICU physicians, core areas were divergent, making different results for other subpopulations regarding EOL survey very likely [11, 26, 27]. Our survey, intended as a point-prevalence study exploring one educational course only, is probably not fully representative. However, since this work was designed as a hypotheses-generating study only, it was not the primary goal to reach comparability or generalizability; instead, by finding out more about novices’ attitudes, it does complete and extend prior work [2, 3], adding valuable information on EOLC on ICUs in Germany from different points of view.

## Conclusions

The present survey underlines a need for improvement in EOLC on German ICUs. Improvement might be achieved by addressing different aspects like quality of life, advanced care planning, continuing EOL education and feedback on outcome data. Different approaches like creating general awareness of the problem (by surveys for example) or by providing tools via specialist societies, already implemented by the German Society of Anesthesiology and Intensive Care Medicine (DGAI) may be helpful [28]. To generally improve EOLC in ICU, therapeutic indications have to be clean-cut, followed by decision making and implementation by the main players, physicians and nurses, patients, their legal representatives and families [3]. Structural changes should first of all include adequate staffing (nurses as well as physicians) and education (including palliative care medicine) in order to ensure high quality support. Whether there are differences or commonalities between patronage and provision and clinical decision-making remains unclear and should be investigated.

## Supplementary Information

**Additional file 1.**

## Data Availability

All data generated or analyzed during this study are included in this article and its supplementary information files. For additional dataset request are available from the corresponding author on reasonable request.
