# Supplementary material for: A beginner’s view of end of life care on German intensive care units
Source: BMC Anesthesiol. 2022 May 18;22:151. doi: 10.1186/s12871-022-01684-8 (PMC9115951; doi:10.1186/s12871-022-01684-8)
Supplement: Supplementary file 1 — Additional file 1. [file 12871_2022_1684_MOESM1_ESM.docx]

**Appendix A Supplementary Structural data (results, discussion, references and questionnaire)**

**Supplementary Results**

Level of care, patronage

Of all complete files, 24 (12%) were delivered by university hospitals and 169 (88%) by non-university hospitals. One participant did not answer this question. Regarding level of care, 42% emanated from primary and secondary level public hospitals, followed by tertiary level (34%) and specialized hospitals (23%). Patronage was confessional in 37%, followed by public (36%) private (21%) and non-profit (4%).

Treatment focus

A “medical” treatment focus was present in 66%, including general internal medicine (48%), cardiologic (15%), and neurological ICUs (3%). A “surgical“ treatment focus was present in 35%, including interdisciplinary/ anaesthesiologic (24%), general surgery (5%), trauma and neurosurgical ICUs (both 2%), and cardiothoracic surgery and urological ICUs (both 1%).

Overall hospital, ICU, IMC and mechanical ventilation capacity

The majority of the participants worked in hospitals with an overall number of 301 – 700 beds (average 42%, 4-75%) and > 700 beds (average 29%, 0-75%). Nearly 2/3 (64%) stated to work in ICUs with ≤ 14 beds, whereas the remaining 36% stated to work on ICUs with > 14 beds. The average number of IMC beds was lower [33% for 0-5 beds (16-63%) and 22% (17-28%) for 6-10 beds]. Mechanical ventilation capacity was 6-10 beds in 27% (17-37%) and 11-15 beds in 21% (8-50%).

Staffing and internal qualification (physicians, nurses, physician/ patient ratio and nurse/ patient ratio)

The number of available specialized intensive care physicians was 0-5 in 59% of all surveyed participants, 5-10 in 20% and 5% were able to provide > 10 intensivists**.** In 54%, between zero (30%) and two (14%) palliative care physicians were available. An average of 34% was unaware about this degree of specialization in their hospitals. Staff qualified as “palliative care nurse” was available in a low number (0-5 nurses, 23%), with a high number of uncertainty (76%). The most common patient / physician ratio ranged from 3-5 (24%) to 6-8 (31%) during regular work hours. In 25%, the ratio was > 10 patients / physician**.** For nursing staff, the majority had a ratio of 2 (21%), 3 (46%) and 4 (12%) patients / nurse.

Additional external services

External palliative care consultants were available in 75% (53-96%), hospital pastoral services in 90% (77-100%) and psychologists in 74% (52-92%) on average. University hospitals provided the highest numbers (palliative care 96%, pastoral services in 100%, psychologists in 92%).

**Supplementary Discussion**

Structural data

The distribution of patronage in our survey resembles actual data from Germany and therefore makes our surveys representative for the distribution of the German hospital landscape [1]. As expected, university hospitals included the highest range of services, but university residents represented only a small proportion of the total study population, which is historically grown throughout the course progression. Of all structural data, only “hospital level of care” and “number of ICU beds” reached statistical significance with a significant higher number of “surgical” residents from maximum care providers attending the course, the latter has grown historically throughout the courses. The higher the share of tertiary care facilities, the higher the average number of ICU beds e. g. owing to the specialization and disease severity of the patients treated [2], so basically seen in this way, the one causes the other. Further structural data is discussed in the supplementary data section.

Additional external services

The availability of additional external services was generally high, with psychologists being the least represented (overall 74%, 52%-92%). As psychological problems on ICUs such as burn-out for medical staff and anxiety for patients and relatives are a matter of concern, one should aim to increase the number of psychological assistances in order to support staff and patients [3].

Staffing and internal qualification (physicians, nurses, physician / patient ratio and nurse / patient ratio)

In line with previous data from Weiss *et al* [4;5], our results revealed still existing discrepancies between the *status quo* and the German interdisciplinary association of intensive care medicine (DIVI) recommendations for physician and nurse/ patient ratio [6]. This is astonishing since German law (PPUGV, Nursing Staff Lower Limits Ordinance) makes a patient/nurse ratio of 2:1 during day and 3:1 during nightshift mandatory from 2021 on [7]. During the peak of the pandemic, the staffing floors were briefly suspended for a few months, but are now active again. However, our pre-pandemic data do not support a proper implementation of this statutory provision, as only 21% were within this ratio. Unfortunately, there is no equivalent for medical services and our data showed a significantly worse ratio, representing a drawback that should be eliminated quickly.

In view of the increasingly strict structural assessments by the Medical Service of German Health Insurances, which make the permanent presence of an ICU doctor mandatory for the reimbursement of ICU medical costs, our data reveal a certain potential for readjustment [8]. A low patient/nurse ratio is as essential as a low patient/physician ratio not only for educational and maintaining reasons but must also be postulated in order to provide reasonable and high-quality EOLC [4]. We completely agree that hospital institutions should have the obligation to ensure time for trained and qualified personal empathically caring for high-quality EOLC [4].

Specialization

There was a high level of uncertainty reflected by an average response rate of “I don`t know” answers in 34% for medical and 76% for nursing specialization, which is most likely owed to the "beginner" status, i.e. the often very limited work experience in the ICU. Nevertheless, the overall proportion of doctors and / or nurses qualified in palliative care medicine appeared to be very low, a finding consistent with prior publications [4]. Estimates of the number of intensive care patients treated annually were also very low, which further supports the "uncertainty hypothesis". Nevertheless, our data may serve as another argument regarding economic burden on medicine as EOLC on ICUs is *eo ipso* personal- and time consuming [4]. Empathy, a key attribute in EOLC, may be difficult to display in face of a steadily rising workload. Besides, despite the relatively pervasive need for EOLC decisions on ICU, there is a high variability between countries, regions and even different ICUs in one hospital regarding decision-making and implementation [9-13]. Following a survey with German residents there seem to be profound knowledge deficits about ethical and legal medical aspects [14], pointing out the necessity to deal with the topic. Already before COVID-19, up to 20% of the population in industrialized nations die during ICU stay, pointing out the importance of EOLC in theoretical and practical education of intensive care beginners [15]. Factors influencing physicians and nurse’s motivation for EOLC include motivation, faith, colleagueship, cooperative conversation, and, last but not least, authentically acting seniors mediating satisfaction at work [16].

Our data complete and extend data from experienced German intensivists [4;5]. Presumably as part of their position, their knowledge of structural data was superior to our collective, impeding a direct comparison. However, as we specially aimed for a beginner´s view of the world (in this case intensive care medicine), we did not expect the participants to know everything in detail.

**Supplementary References:**

1. Federal Statistical Office, Wiesbaden 2016. Gesundheit. Grunddaten der Krankenhaeuser 2015. Fachserie 12 Reihe 6.1.1. Accession Number 2120611157004. 2015. <https://www.destatis.de/DE/Publikationen/Thematisch/Gesundheit/Krankenhaeuser/GrunddatenKrankenhaeuser2120611157004.pdf?__blob=publicationFile>. Accessed 07 Aug 2020.
2. Blum K. Staffing situation in intensive care and intensive medicine. Report of the German Hospital Institute on behalf of the German Hospital Federation. 2017 www. https://www.dki.de/sites/default/files/2019-05/Personalsituation%20in%20der%20Intensivpflege.pdf. Accessed 09. Sep 2021.
3. Parsons LC, Walters MA. Management Strategies in the Intensive Care Unit to Improve Psychosocial Outcomes Crit Care Nurs Clin North Am. 2019;31(4):537-45.
4. Weiss M, Michalsen A, Toenjes A, Porzsolt F, Bein T, Brinkmann A, et al. Structural aspects regarding end-of-life care in German intensive care units managed by anaesthesiologists Anaesth Intensivmed 2018;59:122-31.
5. Weiss M, Michalsen A, Toenjes A, Porzsolt F, Bein T, Theisen M, et al. End-of-life perceptions among physicians in intensive care units managed by anesthesiologists in Germany: a survey about structure, current implementation and deficits. BMC Anesthesiol. 2017 Jul 11;17(1):93.
6. Jorch G, Kluge S, König F, Markewitz A, Notz K, Parvu V. et al. Recommendations on the structure and equipment of intensive care units. Adopted by resolution of the Presidium of the German Interdisciplinary Association for Intensive Care and Emergency Medicine (DIVI) from 30. Nov 2010. <http://www.divi.de/images/Dokumente/Empfehlungen/Strukturempfehlungen/2011_StrukturempfehlungLangversion.pdf>. Accessed 10 Oct 2021.
7. <https://www.bundesgesundheitsministerium.de/personaluntergrenzen.html>. Accessed 02. Aug 2020.
8. [www.rechtsprechung-im-internet.de/jportal/portal/t/b1r/page/bsjrsprod.psml?doc.hl=1&doc.id=KSRE136061518&documentnumber=23&numberofresults=24&doctyp=juris-r&showdoccase=1&doc.part=K&paramfromHL=true#focuspoint](http://www.rechtsprechung-im-internet.de/jportal/portal/t/b1r/page/bsjrsprod.psml?doc.hl=1&doc.id=KSRE136061518&documentnumber=23&numberofresults=24&doctyp=juris-r&showdoccase=1&doc.part=K&paramfromHL=true#focuspoint). Accessed 02 Sep 2020
9. Collins N, Phelan D, Marsh B, Sprung CL. End-of-life care in the intensive care unit: the Irish Ethicus data. Crit Care Resusc 2006;8:315-20.
10. Mark NM, Rayner SG, Lee NJ, Curtis JR. Global variability in withholding and withdrawal of life-sustaining treatment in the intensive care unit: a systematic review. Intensive Care Med 2015;41:1572-85.
11. Sprung CL, Cohen SL, Sjokvist P, Baras M, Bulow HH, Hovilehto S, et al. End-of-life practices in European intensive care units: the Ethicus Study. JAMA 2003;290:790-7.
12. Poulton B, Ridley S, Mackenzie-Ross R, Rizvi S. Variation in end-of-life decision making between critical care consultants. Anaesthesia 2005;60:1101-15.
13. Curtis JR, Engelberg RA, Teno JM. Understanding variability of end-of-life care in the ICU for the elderly. Intensive Care Med 2017;43:94-6.
14. Wandrowski J, Schuster T, Strube W, Steger F. Medical Ethical Knowledge and Moral Attitudes Among Physicians in Bavaria. Dtsch Arztebl Int 2012;109:141-7.
15. Connolly C, Miskolci O, Phelan D, Buggy DJ: End-of-life in the ICU: moving from withdrawal of care‘ to a palliative care, patient-centred approach. Br J Anaesth 2016;117:143-5.
16. Wettig J. What keeps us at work. Dtsch Arztebl 2011; 108(42): A-2247 / B-1895 / C-1875.

**Table 3 Supplementary Material and Methods - Questionnaire**

| Q1 | Category |
| --- | --- |
| Q2 | Level of care |
| Q3 | Patronage |
| Q4 | Treatment focus |
| Q5 | Total number of hospital beds |
| Q6 | Number of ICU/ IMC beds in total |
| Q7 | Number of ICU beds |
| Q8 | Number of IMC beds |
| Q9 | Number of beds with optional ventilation |
| Q10 | Number of ICU/ IMC patients/ year |
| Q11 | Number of ICU patients/ year |
| Q12 | Number of IMC patients/ year |
| Q13 | ICU administration by specialized Intensive Care Physician exclusively placed on ICU |
| Q14 | Minimum of 1 physician exclusively on ICU 24/7 |
| Q15 | 1 physician taking care of n = patients during standardized working hours |
| Q16 | 1 physician taking care of n = patients during night time |
| Q17 | 1 physician taking care of n = patients during weekends/ public holiday |
| Q18 | Number of physicians specialized in Intensive Care |
| Q19 | Number of physicians specialized in Palliative Care |
| Q20 | 1 nurse taking care of n = patients during standardized working hours |
| Q21 | 1 nurse taking care of n = patients during night time |
| Q22 | 1 nurse taking care of n = patients during weekends/ public holiday |
| Q23 | Physicians authorized to train palliative care |
| Q24 | Number of nursing staff allocated to ICU |
| Q25 | Number of nursing staff specialized in Palliative Care |
| Q26 | Palliative Care consultants available in hospital |
| Q27 | Daily counselling available |
| Q28 | Psychologists available on a daily base? |
| Q29 | To which group of physicians do you belong (resident, specialist, staff, consultant) |
| Q30 | I am working in Intensive Care Medicine since |
